# Hospital admissions for respiratory system diseases in adults with intellectual disabilities in Southeast London: a register-based cohort study

**DOI:** 10.1136/bmjopen-2016-014846

**Published:** 2017-03-29

**Authors:** Chin-Kuo Chang, Chih-Yin Chen, Mathew Broadbent, Robert Stewart, Jean O'Hara

**Affiliations:** 1 Department of Psychological Medicine, King's College London (Institute of Psychiatry, Psychology, and Neuroscience), London, UK; 2 South London and Maudsley National Health Service (NHS) Foundation Trust, London, UK; 3 Nursing Department, Chang Jung Christian University, Tainan City, Taiwan; 4 King's Health Partners, South London and Maudsley NHS Foundation Trust and Institute of Psychiatry, Psychology, and Neuroscience, London, UK

**Keywords:** hospitalisation, data linkage, retrospective cohort, mental healthcare, health service utilisation

## Abstract

**Background:**

Intellectual disability (ID) carries a high impact on need for care, health status and premature mortality. Respiratory system diseases contribute a major part of mortality among people with ID, but remain underinvestigated as consequent morbidities.

**Methods:**

Anonymised electronic mental health records from the South London and Maudsley Trust (SLaM) were linked to national acute medical care data. Using retrospective cohort and matched case–control study designs, adults with ID receiving SLaM care between 1 January 2008 and 31 March 2013 were identified and compared with local catchment residents for respiratory system disease admissions. Standardised admission ratios (SARs) were first calculated, followed by a comparison of duration of hospitalisation with respiratory system disease between people with ID and age-matched and gender-matched random counterparts modelled using linear regression. Finally, the risk of readmission for respiratory system disease was analysed using the Cox models.

**Results:**

For the 3138 adults with ID identified in SLaM, the SAR for respiratory system disease admissions was 4.02 (95% CI 3.79 to 4.26). Compared with adults without ID, duration of hospitalisation was significantly longer by 2.34 days (95% CI 0.03 to 4.64) and respiratory system disease readmission was significantly elevated (HR=1.35; 95% CI 1.17 to 1.56) after confounding adjustment.

**Conclusions:**

Respiratory system disease admissions in adults with ID are more frequent, of longer duration and have a higher likelihood of recurring. Development and evaluation of potential interventions to the preventable causes of respiratory diseases should be prioritised.

**Strengths and limitations of this study**

- A nearly population-based dynamic mental disorder cohort in London with a massive data linkage to the national case register system of hospital admission in England.
- Diagnosis of intellectual disability (ID) in secondary mental healthcare was relatively more precise than community studies.
- Data were routinely collected in clinical settings, not particularly generated for research purposes, resulting in missing lifestyle factors (ie, smoking, alcohol intake, physical activity and obesity) for confounding control.
- For the nature of secondary mental health systems, ID patients in adulthood might be underestimated, which limited the generalisability of this study.
- We only focused on hospital admissions of respiratory system diseases with ignorance of milder cases as the outcomes.

## Introduction

Intellectual disability (ID) is defined as a mental disorder with substantially reduced ability to understand new or complex information, to learn new skills and to cope independently, evident during the developmental period in childhood with a lasting effect into adulthood.^1^ The prevalence of ID in England is estimated to be around 2% in adults,^2^ with an estimated 1.07 million people affected and 0.9 million aged 18 years or above in 2013.^3^ Among them, only around 5 per 1000 are known to medical services.^4^

Healthcare of people with ID has been a longstanding concern, with increasingly recognised inequalities in access to healthcare, higher multimorbidity burden, poorer health outcomes and premature mortality.^5–9^ Based on a nationwide hospital discharge register system in Sweden, Auquier *et al*^10^ revealed substantial losses in life expectancy for various mental disorder categories compared with the general population, including 14.7 life years of life lost for adults with ID. A UK population-based study for a specific group of people with moderate to severe ID revealed higher than 8-fold and 17-fold increases in standardised mortality for men and women, respectively.^8^ People with ID have been found to consult GPs more often (82% vs 69%),^11^ and are twice as likely to be admitted into hospital for a physical illness (26% vs 14%) compared with the general population.^12^ Eight per cent of admissions end up as emergency admissions compared with 5% for the general population, and, unlike the general population, emergency admissions occur across the age groups and not predominantly in the very young or elderly.^13^

The Confidential Inquiry into Premature Deaths of People with Learning Disabilities (CIPOLD) reported that 42% of the deaths they reviewed were considered premature and avoidable, either through improved quality of care or effective public health interventions.^14^ The most common reasons were delays or barriers to diagnoses and treatments, although it has been suggested that the inequalities evident in access to healthcare may place many the National Health Service (NHS) Trusts in England in contravention of their legal responsibilities under the Equality Act 2010, the Mental Capacity Act 2005 and the Health and Social Care Act 2008 (Regulated Activities) Regulations 2010.^15^ There is therefore a pressing need to better characterise the pattern of medical service use among people with ID, in order to reduce health inequalities, improve coordination of care for their long-term conditions and reduce the risk of premature death.^16^

Chronic physical conditions, including lung diseases, have been reported more frequently among people with a wide range of mental disorders.^17^ Respiratory system disease has been highlighted as a particularly important physical health issue in people with ID, affecting 46–52% of the ID population, compared with 15–17% in the general population.^18^ In the UK, respiratory system disease was documented as the leading cause in death certifications for people with ID; however, ID itself was rarely recorded in the death certificates.^5^ Although published research to date has focused on people with a wide range of severity for ID, restrictions by small samples, short follow-up periods and limited sample representativeness existed.^19–23^ Specifically, an elevated risk of mortality from bronchopneumonia of around 6.5-fold was reported in a cohort with moderate to severe ID in the UK,^21^ and respiratory infection was found the most common cause of death over a 1-year period in a UK cohort of adults with ID receiving mealtime support for any eating, drinking or swallowing problem.^20^ In a Taiwanese institutional sample of people with ID followed for up to 4 years, pneumonia was the topmost reason for hospitalisation.^19^ Another study in Canada reported a more than twofold adjusted relative risk of asthma admission of people with ID, identified by population-based medical and educational databases, comparing to people without ID.^24^ Issues about knowledge level of inhaled asthma medications and asthma management practice among milder ID patients comorbid with asthma were also assessed by descriptive and qualitative studies in Australia.^22^ ^23^

Therefore, the unsolved research questions are about how ID affects the risks of severe respiratory diseases leading to admission and how much worse their treatment outcomes are by comparing critical indicators for service usage. To address these critical questions concerning the specific usage of healthcare for adults with ID,^11^ ^25^ we carried out a series of analyses in a retrospective cohort of adults with ID known to secondary care mental health services to investigate frequencies of hospitalisation with respiratory system diseases, duration of hospitalisation and risk of readmission, compared with samples within the same catchment area over a follow-up period of up to 5.25 years. Our analysis provided more evidence about the health disadvantages for adults with ID, focusing on respiratory diseases, in order to assess the impact of ID to inform the prevention strategies for this potentially vulnerable group.

## Materials and methods

### Study setting

Details of the study setting are described elsewhere.^26^ In brief, the South London and Maudsley NHS Foundation Trust (SLaM) is one of the largest secondary mental healthcare providers in Western Europe, serving ∼1.36 million residents in southeast London by providing a range of secondary and tertiary mental healthcare services. Since 2006, all clinical records in SLaM services have been converted into electronic form and were made available for research in 2008 by the establishment of Clinical Record Interactive Search (CRIS), an anonymised platform with full clinical information, under the support of the National Institute of Health Research (NIHR). The resulting SLaM Case Register was approved as an anonymised data set for secondary data analyses by the Oxfordshire Research Ethics Committee C (reference number: 08/H0606/71+5). Several studies using the CRIS data resource to investigate physical health consequences of mental disorders have been published.^27–29^

### Study sample

A CRIS search was performed to define people with ID, based on structured WHO ICD-10 diagnostic codes of F70–F79 (mental retardation) entered before the end of the observation period (31 March 2013). This was supplemented by a natural language processing application developed using Generalised Architecture for Text Engineering (GATE) to identify text strings associated with diagnostic statements. The study samples were at least 20 years old at the midpoint of the observation period (1 January 2008–31 March 2013) and had been referred to or were under active review by SLaM services at any time during the period. A pre-existing data linkage was used between CRIS and Hospital Episodes Statistics (HES) data to identify any hospitalisation episode with an associated primary discharge diagnosis of respiratory system disease (ICD-10 J codes). This linked file was generated by the NHS Health and Social Care Information Centre (now NHS Digital), and contains details of all admissions to NHS hospitals in England and Wales for people who are resident in the SLaM catchment, along with a link variable ascertaining their CRIS record, for those who have received SLaM care.

### Statistical analyses

#### Schemes of analyses

With the major interest being ID, there were three schemes of analyses: (1) standardised admission ratios (SARs) during the observation period for any respiratory system disease, and then for each major category of respiratory system disease; (2) duration of hospitalisation, defined as the number of days between admission and discharge dates for the first admission episode (where both dates were available) during the observation period with respiratory system diseases as a primary discharge diagnosis; and (3) risk of readmission with respiratory system disease as a diagnosis following such an admission episode.

#### Standardised admission ratios

In the first comparison, SARs were calculated for people with ID in relation to the SLaM catchment population, with the age and gender structure derived from the 2011 UK Census for the catchment in order to calculate expected admission numbers. SARs were calculated for the observation period from 1 January 2008 to 31 March 2013 for all admissions where respiratory system disease was given as the primary discharge diagnosis and then for each major category of ICD-10 J code, using the number of admissions recorded in HES as the numerator (observed number). The denominator was the expected number of admissions estimated by age-specific, gender-specific and admission year-specific admission rates for the local population multiplied by the age and gender structure in the catchment area given by the 2011 UK Census data. Each age stratum was defined at the midpoint of the observation period and divided into 5-year groups (from 20–24 to 90+). SARs were also calculated by gender and for patient counts by eliminating repeating admissions for J codes in each year.

#### Duration of hospitalisation

In the second set of analyses, for each first admission of an ID case with respiratory system disease in the observation period (1 January 2008 to 31 March 2013), four controls with their first admission of respiratory system disease (ICD-10 J-code) as primary discharge diagnosis were randomly selected from the catchment data, matched by age at admission and gender. Linear regression was used to model the duration of hospitalisation as the outcome, taking into account the matched design by specifying the group id for each group of cases and matched controls in Stata command.

#### Risk of repeat hospitalisation

The third set of analyses was restricted to first hospital admissions in the observation period for people residing in the SLaM catchment. The analysed sample comprised people with respiratory system diseases as a primary discharge diagnosis; the analysis compared those with/without an ID diagnosis using a binary independent variable; and the outcome was a further admission for respiratory system disease after discharge, analysed using the Cox models. The follow-up period was defined by the date of discharge for the index admission as the starting point and date of the next respiratory system disease admission (‘event’ in survival analysis) with 31 March 2013 (end of the observation period) as censoring. Two adjacent hospitalisation episodes within 2 days were considered as one admission and those who died in the index admission were excluded. Ethnicity, discharge method for the index admission (‘clinical advice/clinical consent’, ‘self-discharged/others’ or ‘death’) and number of physical comorbidities other than respiratory system diseases were considered as potential confounders. All analyses were carried out using Stata V.12.1 (StataCorp., College Station, Texas, USA) and an α level of 0.05 was used as the criterion for statistical significance.

## Results

### Study sample characteristics

**Figure 1:**
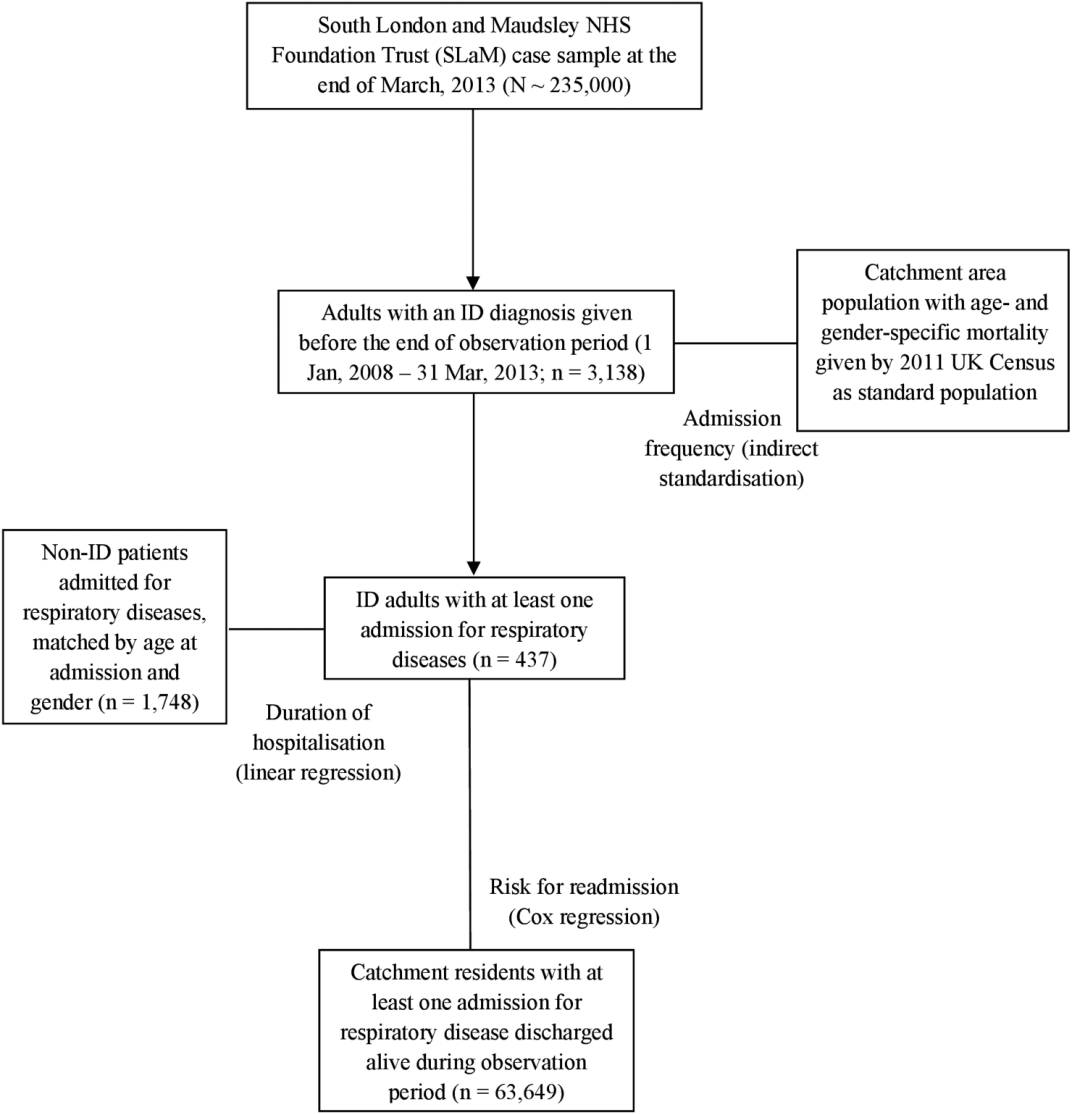
Process for identification of study sample and comparison groups in statistical analysis.

Using the CRIS data resource, a total of 3138 adults with ID aged at least 20 years old were identified, which represented 1.6% of adults on the database; this sample was 55.5% men with a mean age of 44.9 years (SD 17.1). The majority groups were mild (ICD 10 code F70, n=1032; 32.89%) and moderate (F71, n=701; 22.34%) ID cases, followed by other or unspecified ID cases (F78 or F79, n=1087; 34.64%), and then severe or profound cases (F72 or F73, n=318; 10.13%). Ethnicity coding for the majority was white (59.0%), followed by black (23.7%), which broadly reflects the ethnicity profile in the catchment from the 2011 UK Census (55.0% white, 24.7% black).^30^ Figure 1 summarises the groups and data for analysis.

### Standardised admission ratios

Of the 3138 adults with ID identified, 437 (13.9%) had at least 1 admission for respiratory system disease, resulting in 1149 hospitalisation episodes in the observation period with a SAR of 4.02 (95% CI 3.79 to 4.26), further described by gender and subcategory in table 1. Excluding repeat admissions in the same year, SARs were 3.19 overall (95% CI 2.94 to 3.44) and nearly identical for men and women. While most of the raised admission rates were accounted for by lung diseases due to external agents (ICD-10 code: J60–70) with SAR as high as 15.25 (95% CI 11.63 to 19.62; n=60), significant SARs were also found for influenza and pneumonia, chronic lower respiratory diseases and other acute lower respiratory infections.

**Table 1:** Standardised respiratory system disease admission ratios for patients with intellectual disability (N=3138)*

	Standardised admission ratios (95% CI; number of admissions)		
Diagnosis in ICD-10	Total	Male	Female
Diseases of the respiratory system (all J codes)	4.02 (3.79 to 4.26; n=1149)†	4.22 (3.88 to 4.57; n=579)†	3.84 (3.53 to 4.17; n=570)†
Influenza and pneumonia (J09–18)	6.28 (5.46 to 7.19; n=208)†	5.55 (4.52 to 6.73; n=102)†	7.19 (5.89 to 8.70; n=106)†
Other acute lower respiratory infections (J20–22)	6.21 (5.21 to 7.35; n=135)†	6.45 (5.04 to 8.14; n=71)†	5.97 (4.60 to 7.62; n=64)†
Other diseases of upper respiratory tract (J30–39)	0.85 (0.54 to 1.26; n=24)	0.90 (0.52 to 1.47; n=16)	0.76 (0.33 to 1.49; n=8)
Chronic lower respiratory diseases (J40–47)	4.03 (3.74 to 4.33; n=733)†	4.61 (4.15 to 5.11; n=360)†	3.59 (3.24 to 3.98; n=373)†
Lung diseases due to external agents (J60–70)	15.25 (11.63 to 19.62; n=60)†	14.46 (10.13 to 20.01; n=36)†	16.60 (10.64 to 24.70; n=24)†
Other respiratory diseases principally affecting the interstitium (J80-84)	1.34 (0.64 to 2.47; n=10)	1.34 (0.43 to 3.13; n=5)	1.34 (0.44 to 3.14; n=5)
Patient counts for all J codes ‡	3.19 (2.94 to 3.44; n=640)†	3.15 (2.81 to 3.52; n=306) †	3.22 (2.88 to 3.58; n=334)†

*Standard population: residents in London boroughs of Southwark, Croydon, Lambeth and Lewisham in 2011 UK Census.

†Statistical significance.

‡Repeating admissions for respiratory system diseases in the same fiscal year were removed.

### Duration of hospitalisation

Preliminary analyses on the considered factors influencing the duration of hospitalisation for respiratory system diseases by comparing inpatients with ID (n=437) and controls (n=1748) matched by age at admission and gender are shown in table 2. The mean duration of first hospitalisation in the observation period was significantly longer for those with ID (11.72 days, SD=23.86) compared with that for controls (9.08 days, SD=15.90). Ethnicity, discharge method and number of comorbidities also differed significantly between the comparison groups. Table 3 displays the results of univariate and multivariate linear regressions for duration of hospitalisation as the dependent variable. After adjustment for ethnicity, discharge method and number of physical comorbidities in the final linear model, duration of hospitalisation remained significantly longer in the ID group by 2.34 days (95% CI 0.03 to 4.64).

**Table 2:** Descriptive data on people in the first hospitalisation with a respiratory system disease, comparing those with intellectual disability (ID) and age-matched and gender-matched non-ID controls

Variables	Mean±SD/number (%)		p Value *
	Adults with ID (n=437)	Non-ID adults (n=1748)	
Age at admission (years old)	54.59±19.82	54.57±19.81	–
Gender			
Female	232 (53.09)	928 (53.09)	–
Male	205 (46.91)	820 (46.91)	
Length of first hospital stay (days)	11.72±23.86	9.08±15.90	0.027†
Ethnicity			
White	297 (67.96)	1104 (63.16)	<0.001†
Black	45 (10.30)	291 (16.65)	
South Asian	7 (1.60)	56 (3.20)	
East Asian	9 (2.06)	47 (2.69)	
Others/mixed/unknown	79 (18.08)	250 (14.30)	
Discharge method			
Clinical advice/clinical consent	397 (90.85)	1619 (92.62)	<0.001†
Self-discharged/others	10 (2.29)	26 (1.49)	
Death	30 (6.86)	103 (5.89)	
Number of physical comorbidities			
0–2	135 (30.89)	658 (37.64)	<0.001†
3–4	124 (28.38)	442 (25.29)	
5 or more	178 (40.73)	648 (37.07)	

*Dependent t-tests for continuous variables and McNemar's tests for categorical variables.

†Statistical significance.

–, Variables used for matching in study design.

**Table 3:** Linear regression analyses of factors associated with duration of hospitalisation for first admissions with a respiratory system disease, comparing intellectual disability (ID) and age-matched and gender-matched non-ID controls

	B value (95% CI)	
Variables	Separately entered variables (unadjusted)	Simultaneously entered variables (mutually adjusted)
Intellectual disability	2.64 (0.30 to 4.97)*	2.34 (0.03 to 4.64)*
Ethnicity		
White	Ref	Ref
Black	0.25 (−2.49 to 3.00)	1.82 (−0.85 to 4.49)
South Asian	0.03 (−3.64 to 3.69)	−0.45 (−3.68 to 2.78)
East Asian	1.61 (−4.07 to 7.29)	3.02 (−2.22 to 8.26)
Others/mixed/unknown	−3.38 (−4.81 to −1.96)*	−2.18 (−3.55 to −0.81)*
Discharge method		
Clinical advice/clinical consent	Ref	Ref
Self-discharged/others	−5.30 (−7.14 to −3.45)*	−4.84 (−7.19 to −2.48)*
Death	7.91 (4.20 to 11.62)*	3.40 (−0.17 to 6.98)
Number of physical comorbidities		
0–2	Ref	Ref
3–4	2.59 (1.49 to 3.68)*	2.45 (1.28 to 3.62)*
5 or more	12.50 (10.66 to 14.34)*	12.03 (10.08 to 13.99)*

*Statistical significance.

### Risk of readmission

In the analysis of respiratory system disease readmission rate (table 4), after excluding fatalities in the first admission (n=40), a total of 397 adults with ID were identified as having at least 1 admission with a respiratory system disease as a primary discharge diagnosis. Of these, 184 experienced a further hospitalisation for a respiratory system disease, and were compared with 62 286 non-ID counterparts with 23 432 readmissions over the observation period. The unadjusted HR for readmission for respiratory system disease was 1.36 (95% CI 1.18 to 1.57), which was not substantially altered in strength following adjustment for age, gender, ethnicity, discharge method and number of physical comorbidities.

**Table 4:** Cox regression analyses of readmission with respiratory system disease following first hospitalisation, comparing people with/without intellectual disability (N=62 683)

Variables	Mean±SD/number	Proportion of readmission	HR (95% CI)	
			Separately entered variables (unadjusted)	Simultaneously entered variables (mutually adjusted)
Intellectual disability				
No	62 286	37.62%	Ref	Ref
Yes	397	46.35%	1.36 (1.18 to 1.57)*	1.35 (1.17 to 1.56)*
Age at 1st admission (years old)	58.61±20.90	–	1.017 (1.016 to 1.018)*	1.016 (1.015 to 1.016)*
Gender				
Female	34 465	37.09%	Ref	Ref
Male	28 209	38.40%	1.05 (1.02 to 1.07)*	1.03 (1.01 to 1.06)*
Unknown	9	0.00%	–	–
Ethnicity				
White	40 067	41.77%	Ref	Ref
Black	10 043	31.53%	0.71 (0.69 to 0.74)*	0.87 (0.83 to 0.90)*
South Asian	2115	36.45%	0.84 (0.78 to 0.91)*	0.92 (0.86 to 0.99)*
East Asian	1477	29.45%	0.66 (0.60 to 0.72)*	0.77 (0.70 to 0.85)*
Others/mixed/unknown	8981	27.93%	0.62 (0.59 to 0.64)*	0.71 (0.68 to 0.74)*
Discharge method				
Discharged on clinical advice/clinical consent	61 602	37.56%	Ref	Ref
Self-discharged, or by a relative/advocate	1059	44.48%	1.26 (1.15to 1.38)*	1.43 (1.31 to 1.57)*
Others	22	45.45%	1.51 (0.81 to 2.80)	1.64 (0.88 to 3.04)
Number of physical comorbidities				
0–8	21 772	40.58%	Ref	Ref
9 or more	40 911	36.13%	0.79 (0.77 to 0.81)*	0.84 (0.82 to 0.86)*

*Statistical significance.

## Discussion

### Summarised key findings

In a large linked data set covering a defined geographic catchment, we found ID to be associated with increased risk of hospitalisation for respiratory system diseases, increased duration of hospitalisation and increased risk of readmission. More specifically, among the 3138 adults identified with ID from this secondary mental health data resource, a more than fourfold increased frequency of hospitalisation with all respiratory system diseases combined (all J codes in ICD-10) was found based on 1149 episodes recorded during the 63-month observation period. We considered the possibility that a relatively small number of adults with ID might account for most of these admissions; however, after the exclusion of ICD-10 J-code episodes in the same year, the relative risk remained over threefold higher. The average duration of hospitalisation for respiratory system diseases in adults with ID was also longer than their matched counterparts by 2.34 days after adjustment, and a 35% higher risk of readmission with a respiratory system disease was also identified.

### Public health implications

Our analyses provide further evidence on the impact of ID in terms of its medical burden, although more intervention studies are needed to generate prevention strategies specifically for respiratory system diseases in primary and secondary care for this group.^1^ ^14^ ^16^ ^31^ ^32^ Annual health checks have been provided with incentives in primary care for adults with ID in the UK as an intervention resulting in higher general and specific health assessments, and improved detection of new comorbidities such as thyroid and gastrointestinal illnesses through secondary care referrals.^31^ Differing from previously published research,^9^ ^19^ ^20^ ^23–25^ our study has concentrated on ID populations identified by secondary mental healthcare services. Adults whose ID is more likely to be moderate or mild were therefore found to be at a higher risk of respiratory system disease requiring hospital admission. People with severe ID and multiple disabilities were reported at a higher risk of hospital admissions for aspiration pneumonia (J60–70) and premature deaths,^33^ suggesting congenital abnormalities, and diseases of the nervous system and sense organs could be the main underlying problem. Although low birth weight and preterm birth have been well characterised as underlying lung diseases in children with ID,^33^ further research is required on factors accounting for increased respiratory system disease in adults. For this cohort, respiratory infection was a major cause of admission, which might well be amenable to early intervention and public health strategies such as influenza vaccination.

### Mental healthcare implications

The study cohort consisted of adults with ID; a secondary mental healthcare provider might be selectively choose those with less severe levels of ID and higher psychiatric comorbidities. Traditionally, delays in accessing physical healthcare were thought to be largely due to difficulties recognising symptoms by the individual with ID or their carers. However, the CIPOLD in the UK declared that while adults with ID presented to healthcare services, there were delays in diagnosis and treatment.^14^ Since the prevalence of adulthood ID is reported around 2% in England,^2^ the catchment area for SLaM has 1 369 048 residents older than 20 years (2011 UK Census data) and thus can be expected to contain 27 381 individuals with ID. The prevalence of mental disorders in the adult ID population is reported to be between 20% and 40%, depending on in the inclusion of ‘challenging behaviours’.^34^ Thus, the cohort size of 3138 individuals on our CRIS database would suggest that a significant number of adults with ID are either not known to, or not identified by, mental health services. Adults with ID involved in our analysis were limited to the ones with mild to moderate ID and may be comorbid with other mental disorders, which restricted the generalisability of our outcomes to people with ID with a wide range of severity. However, since the ID cases in our analyses were presumably milder than the whole ID population and we could reasonably envisage that the situation of respiratory system diseases admission is worse along with the severity of ID, the significant results revealed in our analyses might have been rather conservative and the real effect sizes should be even bigger.

### Potential solutions for improvement

Enhancement of education of doctors and nurses with more ID health content in their curriculum might be of most importance to improved healthcare for people with ID.^35^ ^36^ However, a survey in 2006 of six European countries (Finland, Germany, the Netherlands, Norway, Sweden and the UK) and Australia, Canada, Japan and the USA reported that attention to the specific health aspects of ID in the total education programme for medical students varied from 0 to 36 hours. In most countries, there is no specific attention, and just general information spread over Psychiatry, Paediatrics and Medical Genetics.^37^ Actions have been taken in the UK to redesign the curriculum of undergraduate medical education to address the issues about health inequalities for people with ID, in order to increase knowledge, skills and reducing stigmatisation.^38^ To certain extent, small-scale intervention studies in primary care settings had shown that feasible health checks might be able to meet health monitoring needs and will be cost-efficient for adults with ID in Scotland.^39^ ^40^ Besides, the effect of an interdisciplinary, guideline-based continuing education course for primary care professionals to the care of adults with ID was assessed in Canada. Significant improvement was identified in terms of the frequency of guideline use, assessment of behaviour change, comfort level in caring for people with ID and related knowledge.^41^ The development of clinical guidelines for this particular group of people, covering issues about promoting the integration of healthcare services provided by primary and secondary healthcare systems, health screening and continuing education, might be of greater importance, too.^42^

### Strengths and weaknesses of this research

This research benefited by a large database from a near-monopoly secondary mental healthcare provider in southeast London with a linkage to national hospitalisation data on physical disorders, clinical diagnoses of ID given by ID specialists working in secondary mental healthcare, and a cohort study design. Nonetheless, some important limitations require consideration. First, both the databases analysed were established for the purpose of administration and/or routine clinical practice, rather than research, resulting in incomplete data including those on lifestyle factors such as smoking, alcohol intake, physical activity and obesity, which might be important potential confounders. Second, while the number of adults with ID identified in our mental health case register is a recognisable and clinically relevant subset, it is likely to be a marked underestimate of the population with ID; thus, the generalisability of our current analysis should be viewed with caution. However, as what was further discussed in a previous paragraph, the potentially conservative estimations shifting to null (but still statistically significant) made our analysis outcomes even more reliable. Third, although we identified 3138 adults with ID and 640 of them had had at least 1 admission because of respiratory system diseases, the statistical power for further analyses on the duration of hospitalisation and risk of readmission for all causes of respiratory diseases as a whole was only barely enough to show significance. So, we were not allowed to look into specific respiratory system diseases because of limited statistical power.

### Concluding summary and directions of future research

Drawing a conclusion, people with ID accessing secondary mental healthcare services were at a higher risk of respiratory system diseases requiring hospitalisation compared with the local general population, including respiratory infections and chronic lower respiratory disease. Once admitted, they had longer durations of hospitalisation and were more likely to be readmitted with a diagnosis of respiratory system disease. This suggests early diagnosis and interventions, public health strategies and lifestyle choices may be important in addressing their health inequalities and poorer outcomes. Further studies assessing the effect of potential prevention strategy (eg, influenza vaccination) in the target population in secondary mental healthcare setting are warranted. Besides, further research focusing on the evidence of delays or barriers to diagnoses and treatments for physical illness among people with ID, identifying healthcare needs and accessing appropriate care in response to need changing is also of great research interests.
